# Tinnitus and tinnitus disorder: Genetic, neurobiological, and clinical differentiation

**DOI:** 10.1016/j.isci.2026.116080

**Published:** 2026-06-03

**Authors:** Dirk De Ridder, Tobias Kleinjung, Jae-Jin Song, Divya Adhia, Matt Hall, Anusha Yasoda-Mohan, Sven Vanneste, Alain Londero, Nathan Weisz, Winfred Schlee, Ana Belen Elgoyhen, Christopher Cederroth, Jose Antonio Lopez-Escamez, Silvano Gallus, Stefan Schoisswohl, William Sedley, Grant Searchfield, Shi Nae Park, Berthold Langguth

**Affiliations:** 1 Section of Neurosurgery, Department of Surgical Sciences, University of Otago, Dunedin, New Zealand; 2 Department of Otorhinolaryngology, Head and Neck Surgery, University Hospital Zurich, University of Zurich, Swiss Federal Institute of Technology Zurich, Zurich, Switzerland; 3 Department of Otorhinolaryngology, Seoul National University Bundang Hospital, Seongnam, Republic of Korea; 4 Global Brain Health Institute of Neuroscience, Trinity College Dublin, Dublin, Ireland; 5 Université Paris Cité, Institut Pasteur, AP-HP, Hôpital Lariboisière, Service ORL, INSERM, Fondation Pour l'Audition, IHU reConnect, Paris, France; 6 Centre for Cognitive Neuroscience, Department of Psychology, University of Salzburg, Salzburg, Salzburg, Austria; 7 Institute for Information and Process Management, Eastern Switzerland University of Applied Sciences, St. Gallen, Switzerland; 8 Department of Pharmacology, University of Buenos Aires, Buenos Aires, Argentina; 9 Laboratory of Experimental Audiology, Department of Physiology and Pharmacology, Karolinska Institute, Stockholm, Sweden; 10 The University of Sydney, School of Medical Sciences, Faculty of Medicine and Health, Sydney, NSW, Australia; 11 Department of Medical Epidemiology, Istituto di Ricerche Farmacologiche Mario Negri IRCCS, Milan, Italy; 12 Department of Psychiatry and Psychotherapy, University of Regensburg at Bezirksklinikum Regensburg, Regensburg, Germany; 13 Translational and Clinical Research Institute, Newcastle University, Newcastle, UK; 14 Audiology, University of Auckland, Auckland, New Zealand; 15 Department of Otolaryngology-HNS, Seoul St. Mary’s Hospital, The Catholic University of Korea, Seoul, Republic of Korea

**Keywords:** psychiatry, genetics, behavioral neuroscience, clinical neuroscience

## Abstract

Tinnitus is the conscious perception of sound in the absence of an external acoustic source. When accompanied by emotional distress, cognitive dysfunction, or autonomic arousal leading to behavioral and functional impairment, it is termed “tinnitus disorder.” This perspective synthesizes genetic, epidemiological, and neuroimaging evidence supporting the distinction between tinnitus and tinnitus disorder. Genetic studies indicate that tinnitus is linked to multiple common variants of small effect size, whereas tinnitus disorder involves rarer variants exerting larger effects. Epidemiologically, hearing loss is the primary risk factor for tinnitus, whereas personality traits like neuroticism, mood, and sleep disturbances predict tinnitus disorder. Neuroimaging identifies three interrelated neural pathways: a lateral “loudness” pathway, a descending “inhibitory” pathway, and a medial “distress” pathway that is specifically activated in tinnitus disorder, providing a neural basis for tinnitus related suffering. Future needs include the establishment of standardized diagnostic criteria and a severity grading system of tinnitus disorder.

## Graphical abstract

**Figure:**
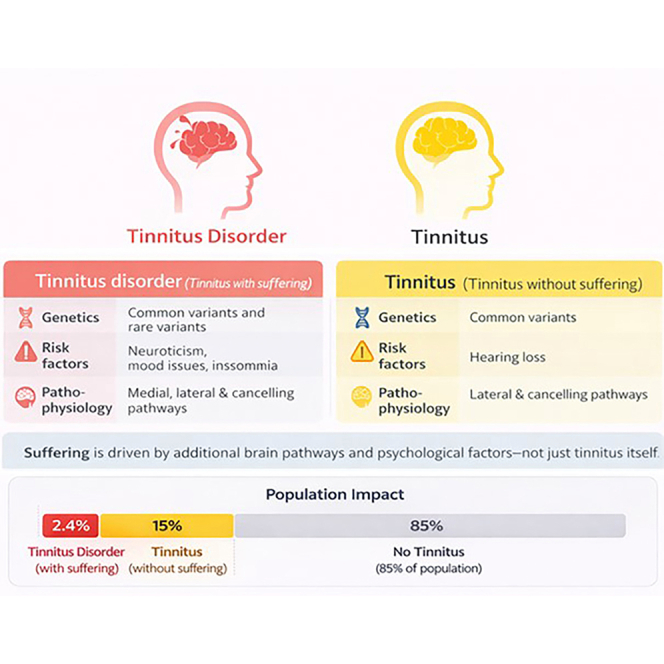

## Introduction

This perspective article describes genetic, pathophysiological, epidemiological, neuro-imaging and clinical support for a categorical distinction between the sound component of tinnitus and the suffering and/or disability component that accompanies tinnitus in about 20% of the tinnitus population. This perspective offers a categorical approach rather than a dimensional approach, not only because the available literature supports this approach but also due to its easier implementation and greater pragmatism in clinical practice currently.

### Tinnitus and tinnitus disorder

Tinnitus has been defined as the conscious awareness of a tonal or composite noise for which there is no identifiable corresponding external acoustic source.^1^

When tinnitus is associated with emotional distress, cognitive dysfunction, and/or autonomic arousal, leading to behavioral changes and functional disability, it is referred to as “tinnitus disorder.”^1^ In other words, “tinnitus” describes the auditory or sensory component, whereas “tinnitus disorder” reflects the auditory component and the associated perceived suffering.^1^ Acute tinnitus is often considered a symptom of an underlying condition such as an infection, trauma, or sudden hearing loss. In contrast, chronic tinnitus is defined as the presence of tinnitus for more than three months, regardless of the cause, and is increasingly being recognized as a health condition in its own right rather than merely a symptom of another disease.^1^ This is similar to what has been described for pain, the somatosensory analogue of tinnitus.^2^ Tinnitus shares similarities with pain with regards to definition, pathophysiology, clinical presentation, and treatment.^2^^,^^3^^,^^4^^,^^5^^,^^6^^,^^7^^,^^8^^,^^9^^,^^10^

### Tinnitus: Categorical versus dimensional approach

The current article conceptualizes tinnitus as a categorical entity rather than a dimensional one, even though some publications advocate dimensional approaches.^11^^,^^12^ The reason is pragmatic, in that a categorical approach has advantages in clinical practice: it is easy for diagnosis and communication, e.g., to fit in ICD and DSM classifications,^1^ easy for clinical decision-making, and is in keeping with traditional medical thinking. The disadvantage is that the boundaries are artificial, some patients may not fall into the created categories, and categories do not capture the gradual severity differences. By adding categories, e.g., grading, in tinnitus disorder, the latter disadvantages can partially be overcame, but dimensional approaches also have advantages,^13^^,^^14^^,^^15^ as they capture individual variability better and permit optimized individualized treatment.

### Tinnitus as a global health problem

According to a recent meta-analysis, the global prevalence of chronic tinnitus is estimated at 14.4%. Severe tinnitus, which can be considered a proxy for tinnitus disorder, occurs in 2.3% of the population.^16^ The incidence of tinnitus is 1,164 per 100,000 person-years.^16^ Tinnitus is evenly distributed among sexes,^16^ but the suffering, i.e., tinnitus disorder, seems to be higher in women.^17^^,^^18^^,^^19^^,^^20^^,^^21^ Chronic tinnitus is a major health problem worldwide, affecting 740 million people, of which 120 million exhibit tinnitus disorder.^16^ This suffering consists of tinnitus-associated stress,^22^ depression,^23^^,^^24^ anxiety,^24^ and sleep problems,^25^^,^^26^ sometimes even leading to suicidal ideation, attempts, and completed suicide.^27^^,^^28^ Chronic tinnitus is also associated with cognitive dysfunction, affecting attention, learning, memory, and decision-making.^29^ With its high prevalence, chronicity, and major impact on emotion, cognition, and behavior of affected individuals, tinnitus and tinnitus disorder also constitute a major socioeconomic problem. A systematic review reported the mean annual estimates per patient ranging between EUR 1,544 and EUR 3,429 for healthcare costs, between EUR 69 and EUR 115 for patient and family costs, and between EUR 2,565 and EUR 3,702 for indirect costs, including productivity loss.^30^ A Swedish cohort study found a more than 3-fold increased risk of disability pension for individuals with sick leave due to tinnitus or hearing disorders,^31^ and a recent European study revealed the yearly mean out-of-pocket expenses of patients with slight, moderate, and severe tinnitus as EUR 368, EUR 728, and EUR 1,492, respectively.^32^ In the US, disability compensation for tinnitus among military personnel was over 1 billion dollars in 2020 and is expected to grow further.^33^ Since patients seek care from health care providers because they are suffering from tinnitus symptoms, these costs likely reflect primarily the costs associated with tinnitus disorder.

## From an operational to a theoretical definition of tinnitus

An international operational definition of tinnitus has been developed,^1^ with a similar alternative version in France,^34^ but no theoretical definition yet exists. An operational definition describes in a pragmatic way what is meant by a certain term, such that the term is concrete, unambiguous, and measurable during clinical diagnosis and treatment.^1^ Operational definitions can change with new insights. Theoretical definitions, on the other hand, aim to define disease entities, which can be delineated from each other by differences in etiology, pathophysiology, and clinical manifestation. A theoretical definition is derived from a theory that includes causal predictions and that can change based on new evidence.^1^ In this article, we summarize available evidence (genetic, pathophysiological, and clinical) for the distinction between tinnitus and tinnitus disorder. In other words, we provide support that the proposed operational definition also qualifies as a theoretic definition for tinnitus and tinnitus disorder.

## Genetic structure differs between tinnitus and tinnitus disorder

**Key concept box 1: Key concept box 1 explains the most important terms to help better understanding of the section on genetic principles**

**Common genetic variants**

Common genetic variants, often called common polymorphisms, are DNA changes like single nucleotide polymorphisms (SNPs) that occur at high frequencies in a population, typically with a minor allele frequency (MAF) of >0.05 or more. These variants are widespread, have been easily detected in genome-wide association studies (GWASs), and usually have smaller effects on traits or diseases.

**Rare genetic variants**

Rare variants occur at low frequencies, generally defined by a MAF below <0.05, and are often more recent mutations subject to purifying selection. They tend to have larger functional impacts and require massive parallel sequencing (genome or exome sequencing) for detection because genotyping chips miss them.

“Heritability”: proportion of the “variation” in a trait within a population that can be attributed to genetic differences between individuals, rather than environmental factors.

“Effect size”: effect size of a genetic variant measures the magnitude of its influence on a trait or disease risk, typically quantified as the regression coefficient (β) in linear or logistic regression models.

“Phenotype”: physical features (like height or eye color), biochemical properties, physiological functions, and behaviors that can be measured or observed, like tinnitus.

Tinnitus heritability is supported by twins, adoptees, and familial aggregation studies.^35^^,^^36^^,^^37^ GWASs have shown few common variants with a small effect size on tinnitus phenotype.^37^^,^^38^ These studies have several limitations, including that the phenotype was heterogeneous and self-reported and limited to European ancestry. Conversely, rare variant analysis and gene burden tests have identified several genes showing an excess of missense variant associated with tinnitus extreme phenotype,^37^^,^^39^ a surrogate of tinnitus disorder. Rare variants are present in few individuals in the tail of the phenotype distribution, but they have a large effect size on a narrower and more precise phenotype and offer an alternative approach to identify candidate genes for further research and validation.^37^^,^^39^

Tinnitus phenotype is considered an umbrella phenotype that includes tinnitus disorder, which is a narrow phenotype. Comparing the genetic structure of a broad vs. an endophenotype included in the umbrella is not the best approach. This would be similar to comparing any type of hearing loss, including age-related hearing loss (common disorder) with congenital, profound hearing loss (rare disorder), as the former is a heterogeneous condition associated with common variants and the latter is associated with a few genes leading to a severe hearing disorder.

We consider that comparing both extremes of the phenotype distribution (mild vs. severe phenotype) using gene burden tests would be a more logical approach, but this will require a large sample size with deep phenotype data, controlling for ancestry and an external population-specific reference dataset.

Hearing loss is a significant risk factor for tinnitus, with both conditions sharing numerous genetic variants. Additionally, there are specific genetic variants that are unique to tinnitus (Clifford 2024).

Multiple epidemiological studies in large cohorts of individuals with tinnitus, including twins,^36^^,^^40^ adoptees,^41^ and familial aggregation studies^42^ have reported evidence to support a significant tinnitus heritability (31%–43%), particularly for severe bilateral tinnitus,^35^^,^^40^^,^^42^ i.e., in what now would be defined as tinnitus disorder. This heritability is explained by genetic variation, and different genes are associated with tinnitus and tinnitus disorder. These genes involve 7 different pathophysiological relevant pathways: (1) neural activity (*BDNF*, *GDNF*, *ANK2*, *GPM6A*, *NAV2*, *TMEM132D*, and *RCOR1*), (2) neurotransmission (*SLC6A4*, *GRM7*, and *GBRB3*), (3) metabolism (*AKAP9*, *COMT*, and *ACE*), (4) ion channels (*CACNA1E*, *ADD1*, *KCNQ1*, and *KCNE1*) (5) inflammation (*TSC2* and *TNFRSF1A*), (6) structural genes (*WDPCP*), and (7) mitochondrial genes related to hearing loss.^37^

According to these findings, tinnitus and tinnitus disorder differ not only in their risk genes but also in their respective effect sizes on the phenotype.^37^^,^^39^^,^^43^ Rare genetic variants associated with tinnitus disorder involve those related to neural activity (e.g., *ANK2*, *NAV2*, and *TMEM132D*), inflammation (e.g., *TSC2*), metabolism (e.g., *AKAP9*), and calcium channel function (*CACNA1E*),^37^^,^^39^ and they have a large effect size on the phenotype. These genes differ from genes for which common variants have been found in tinnitus GWASs, highlighting distinct genetic underpinnings for tinnitus disorder compared with tinnitus alone.^44^ Common variants may have pleiotropic effects and can be associated with different phenotypes, and this could explain the frequent association of tinnitus with other conditions such as hearing loss, hyperacusis, headache, or anxiety. Of note, some of the genes related to tinnitus disorder such as *TMEM132* have also been associated with anxiety,^45^^,^^46^ providing one possible mechanism for the co-occurrence of tinnitus disorder and anxiety.

A key question remains: how these genetic differences contribute to the development of tinnitus and tinnitus disorder? A polygenic model combining common and rare variants with different effect sizes on the tinnitus phenotype could explain the multidimensional structure of tinnitus disorder (autonomic, cognition, emotion, and perception) and the expressivity range in the phenotype, where disability could be linked to one or more of these dimensions.

**Figure 1:**
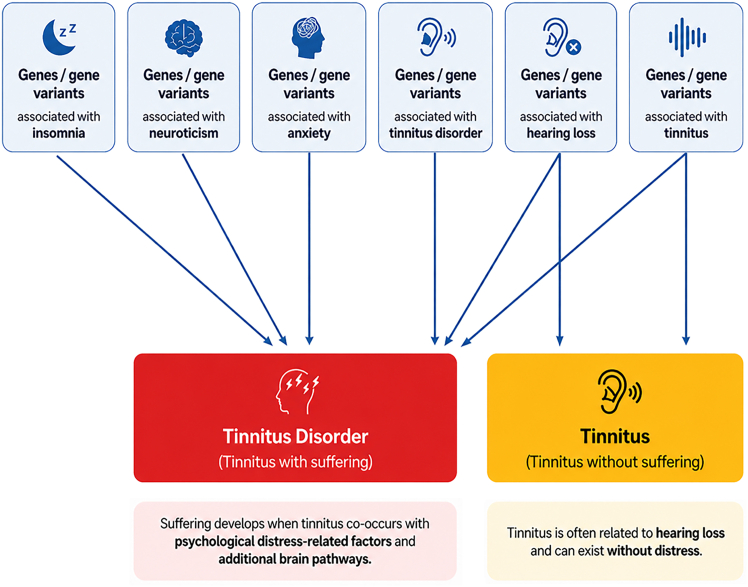
Schematic illustration of direct and indirect genetic influences on tinnitus and tinnitus disorder

Furthermore, risk factors for tinnitus disorder such as neuroticism^47^^,^^48^ or insomnia,^49^^,^^50^^,^^51^ increase the likelihood of people with tinnitus to follow the chronic suffering trajectory. Neuroticism is indeed a risk factor for chronic grief/di5t=^52^ and tinnitus distress.^48^^,^^53^^,^^54^^,^^55^ Some genetic variants associated with neuroticism overlap with the genes involved in resilience, and resilience, in turn, influences whether tinnitus progresses to tinnitus disorder in an affected individual.^56^ Considering that neuroticism genes also overlap with anxiety and depression risk genes,^44^ this may explain why people with genetic risk genes for neuroticism may have an increased risk of developing tinnitus disorder.^47^^,^^48^^,^^53^^,^^57^ Insomnia is also a risk factor for development of burdensome tinnitus. Indeed, insomnia can lead to immune dysfunction,^58^^,^^59^^,^^60^ triggering a long-term neuroinflammatory pathology, which is known to be involved in the development and chronification of tinnitus.^61^^,^^62^^,^^63^^,^^64^ Furthermore, low-grade neuroinflammation is also associated with anxiety,^61^ depression,^65^^,^^66^ and posttraumatic stress disorder (PTSD),^67^^,^^68^^,^^69^ i.e., comorbidities, which turn tinnitus into tinnitus disorder. While cytokine production is increased in tinnitus,^64^ not all inflammatory proteins, e.g., FGF-21, MCP4, GDNF, CXCL9, and MCP-1 exhibit increased levels.^70^ For some risk factors, a bidirectional relation may exist. For example, anxiety, depression, and insomnia may predate and potentially trigger tinnitus,^71^^,^^72^ but tinnitus itself, for example, in the setting of neuroticism, may trigger anxiety, insomnia, and depression.^73^ Based on a large-scale study that looked at risk factors predicting the development of tinnitus disorder 9 years later, hearing loss, neuroticism, sleep problems with fatigue, and life stressors emerged as key factors.^55^ Intriguingly, these risk factors are the same that predict whether someone with pain will develop a pain disorder when replacing hearing loss with overweight (Figure 1).^74^

## Brain pathways involved in tinnitus and tinnitus disorder

### The lateral loudness pathway, the medial distress or suffering pathway, and the descending inhibitory pathway

**Figure 2:**
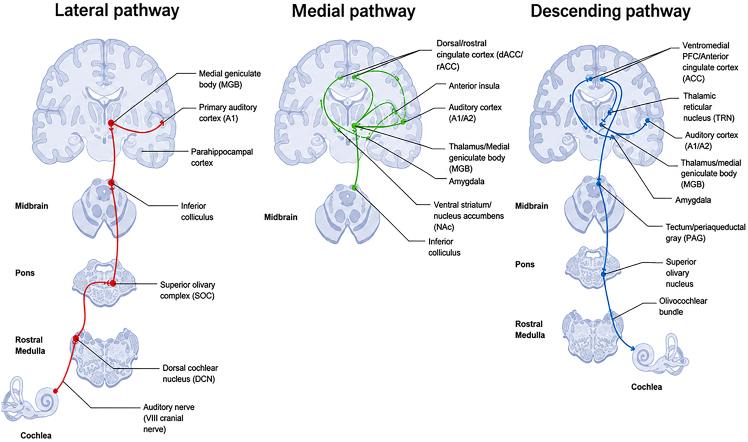
Theoretical model of anatomical pathways associated with 3 different aspects of tinnitus based on functional imaging The lateral pathway conveys perceptual aspects of tinnitus, the medial pathway processes tinnitus suffering, and the descending pathway can be considered as an inhibitory “noise canceling” pathway that determines, for example, fluctuations of tinnitus over time. Based on the proposed differentiation between “tinnitus” and “tinnitus disorder,” “tinnitus” is characterized by a balance between lateral and noise canceling pathway, whereas “tinnitus disorder” is characterized by co-activation of the medial pathway.

Auditory information is processed in two pathways,: classical (also known as lemniscal pathway), and non-classical (also known as extralemniscal pathway). The activity in these pathways is balanced by a third inhibitory pathway (Figure 2). It has been shown that an auditory stimulus reaches consciousness only if the dorsal anterior cingulate cortex (dACC) and insula of the medial pathway (=salience network) are co-activated.^75^ Thus, exactly the same stimulus intensity can either be consciously perceived or not perceived, depending on the co-activation of the salience network, analogous to what is described in pain.^76^ This may relate to how much the noise canceling network can be activated,^77^^,^^78^ which determines for how much time and how loud an auditory stimulus like tinnitus is perceived,^79^ also analogous to pain.^80^

Consequently, it has been proposed that the conscious percept of an auditory stimulus depends on a balance between the activity in the lateral plus medial network mobilization versus the noise canceling system.^8^ This can be computed in a simple way by calculating the ratio of current density (=power) of activity in the auditory cortex as a proxy of the lateral system, the dACC as a proxy of the medial system, and the pregenual anterior cingulate cortex (pgACC) as a proxy for the noise canceling system,^8^ also analogous to what has been described for pain.^81^^,^^82^^,^^83^^,^^84^ Other approaches looking at a balance between the Bayesian inference network and the default mode network (DMN) have also been employed to distinguish between tinnitus and no tinnitus.^85^

The classical auditory pathway is specific for the auditory modality and phylogenetically more recent, whereas the non-classical auditory pathway is multimodal and phylogenetically older.^86^^,^^87^ The lemniscal, or core thalamocortical projection, carries tonotopically organized and auditory specific information and forms the lateral auditory system.^8^ In contrast, the extralemniscal thalamocortical pathway plays an important role in multisensory integration, temporal pattern recognition, and certain forms of learning^88^ and is densely connected with the midbrain and cortical and limbic-related sites, thereby constituting the medial tinnitus pathway. This pathway involves the rostral-to-dorsal anterior cingulate (rdACC) and anterior insular cortex.^8^^,^^89^ These regions have been implicated in salience-, stress-, autonomic-, and distress-related processing^81^^,^^90^^,^^91^^,^^92^ across multiple paradigms. However, such functional interpretations should be made with caution, as assigning specific psychological functions to regional activations relies, in part, on reverse inference and is, therefore, not specific. The medial pathway in sensory processing is coined “medial” because the auditory and somatosensory fibers of this separable pathway relay in the medial nuclei of the thalamus on the trajectory to the anterior insula and rdACC. It overlaps anatomically with the salience network in cognitive neuroscience^8^^,^^81^ and the stress network in affective neuroscience.^81^

Autonomic nervous system processing in tinnitus has been linked to the insula, subgenual anterior cingulate cortex (sgACC), and dACC, using simultaneous heart rate variability (HRV) and electroencephalography (EEG) recordings^93^^,^^94^ and overlap with a general (dis)stress network,^91^^,^^95^ also present in tinnitus distress.^96^^,^^97^^,^^98^ These area overlaps also overlap with the medial pathway and salience network and are in agreement with areas of central autonomic control, as identified by a functional magnetic resonance imaging (fMRI) meta-analysis.^99^

Empirical support for differential involvement of the lateral pathway in “tinnitus” and the medial pathway in “tinnitus disorder” comes from a recent study, which aimed at the identification of neurophysiological markers for tinnitus presence and severity.^100^ In this study, tinnitus patients differed from controls in the activity in central auditory pathways, independent of tinnitus severity (lateral pathway). In contrast, tinnitus severity was associated with signatures of affective and autonomic processing (pupil dilations and facial movements elicited by emotionally evocative sounds), presumably mediated via the medial pathway.

The two tinnitus provoking pathways are balanced by a descending tinnitus inhibitory pathway,^77^^,^^78^^,^^101^ involving the sgACC,^77^ pgACC,^102^ and rostral anterior cingulate cortex (rACC).^103^ This connects to the tectal longitudinal column^104^^,^^105^ and further relays to the olivocochlear bundle,^106^ the main efferent auditory bundle that connects to the cochlea.^107^ This descending inhibitory auditory pathway lies adjacent to the descending pain inhibitory pathway.^105^

The descending tinnitus inhibitory pathway reflects the capacity of the brain to suppress acute or ongoing tinnitus,^78^^,^^108^ and it can be assumed that depending on the function of the descending system, tinnitus can be constant, fluctuating, or absent.^79^^,^^102^

### Neuroimaging of tinnitus and tinnitus disorder

**Figure 3:**
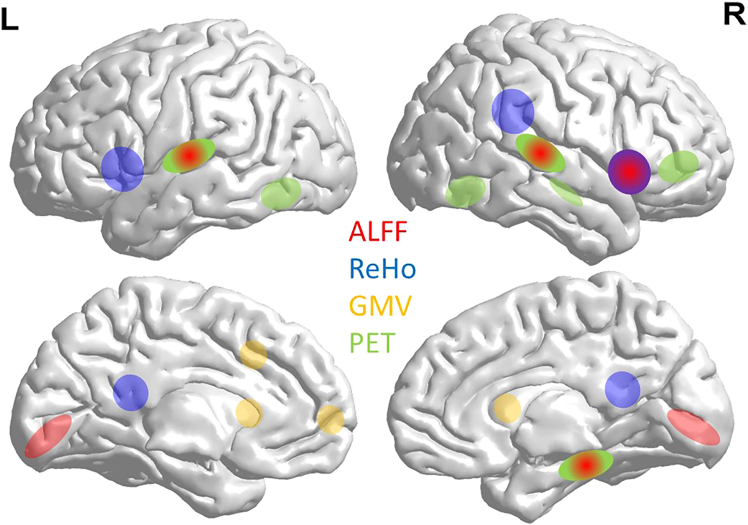
Summary of meta-analyses of structural and functional (PET and fMRI) imaging in tinnitus, based on meta-analyses^109^^,^^110^^,^^112^ ALFF, amplitude of low-frequency fluctuations; ReHo, regional homogeneity; GMV, gray matter volume (based on structural MRI); PET, metabolic activity based on positron emission tomography; fMRI, functional magnetic resonance imaging.

Systematic reviews of neuroimaging studies have found neural correlates of tinnitus within and outside the auditory network (Elgoyhen et al. 2015). A meta-analysis based on gray matter volume (GMV) changes in structural MRI revealed alterations in the caudate nucleus, supplementary motor area, and ventromedial prefrontal cortex.^109^ A functional imaging meta-analysis focusing on resting state brain abnormalities such as amplitude of low-frequency fluctuations (ALFF) identified activity changes in the auditory cortex, insula, and parahippocampus but also in the visual cortex.^110^ The same meta-analysis also looked at regional homogeneity (ReHo) and found changes in the insula, temporoparietal junction, and posterior cingulate cortex, which was confirmed by another meta-analysis.^111^ A meta-analysis of positron emission tomography (PET) scans found metabolic changes in the temporooccipital junction, auditory cortex, parahippocampus, dorsolateral prefrontal cortex (DLPFC), and midtemporal cortex.^112^ It should be noted that most imaging studies did compare people with and without tinnitus but not differentiate between tinnitus patients without and with suffering. However, as most samples were recruited among patients presenting at tinnitus clinics, it can be assumed that the majority had bothersome tinnitus, which could be defined as tinnitus disorder. But, as mentioned above, no thresholds or grading have yet been developed and agreed upon. Many studies did specifically look at the distress or suffering component^94^^,^^96^^,^^98^^,^^108^^,^^113^^,^^114^^,^^115^^,^^116^^,^^117^^,^^118^^,^^119^^,^^120^ and implicate especially the salience network (=medial pathway) and posterior cingulate cortex (PCC) of DMN in suffering/distress. At the same time, these interpretations remain necessarily tentative: neuroimaging findings in regions such as the anterior cingulate, insula, or parahippocampus are not uniquely diagnostic of “suffering” or “salience,” but rather are consistent with broader domain-general processes that may contribute to tinnitus-related distress. But counterintuitively also the auditory cortex is implicated in distress,^113^^,^^121^ which could be explained by the fact that loudness and distress correlate, albeit in a non-linear way.^120^^,^^122^ As a result, statistical analyses of imaging studies may also correlate tinnitus distress to the auditory cortex (Figure 3).

An increasing number of neuroimaging studies have focused on brain alterations that are related to tinnitus distress (Table 1), revealing differences in brain structure, brain activity, and brain connectivity. Most of these studies have analyzed correlations with tinnitus severity, as measured by the score of a tinnitus questionnaire, but some have also contrasted groups with high and low tinnitus distress.

**Table 1:** Neuroimaging studies focusing on tinnitus distress

Author	Method	Brain areas	Connections	Comments
Schlee et al. 2008^123^	MEG auditory evoked	–	ACC-R frontal lobe ACC-R parietal lobe	correlation with tinnitus distress
Schlee et al. 2009^124^	MEG	–	temporal cortex to prefrontal cortex, orbitofrontal cortex, and parieto-occipital cortex	correlation with tinnitus distress
Vanneste et al. 2010^98^	QEEG	increased alpha activity in scACC, insula, parahippocampus, and amygdala; reduced alpha activity in posterior cingulate cortex, precuneus, and DLPFC	–	high-distress tinnitus patients compared to low-distress tinnitus patients
Maudoux et al. 2012^125^	rs fMRI	posterior cingulate/precuneus region	connectivity of the posterior cingulate/precuneus region	correlation with tinnitus distress
Schecklmann et al. 2013^113^	FDG PET	L and R posterior inferior temporal gyrus L and R post parahippocampal interface	–	correlation with tinnitus distress
Golm et al. 2013^126^	fMRI (presentation of sentences with tinnitus-related content)	L MFG	fronto-parietal-cingulate network	high-distress tinnitus patients compared to low-distress tinnitus patients
Schecklmann et al. 2013^121^	VBM	negative with L and R auditory cortex and insula	–	correlation with tinnitus distress
Chen et al. 2014^127^	rs fMRI	R middle temporal gyrus R superior frontal gyrus	–	increased amplitude of low-frequency fluctuations correlates with tinnitus distress
Vanneste et al. 2015^128^	QEEG	activity in sgACC, dACC, and postACC	auditory cortex with sgACC, dACC, and postACC	correlation with tinnitus distress
Chen et al. 2015^129^	rs fMRI	–	L ant insula with L MFG; R ant insula with R MFG;	correlation with tinnitus distress
Carpenter-Thompson et al. 2015^130^	fMRI (presentation of affective sounds)	higher activation of amygdala and parahippocampus; lower activation of frontal regions	–	high-distress tinnitus patients compared to low-distress tinnitus patients
Vanneste et al. 2015^128^	MRI (VBM)	cerebellum	–	correlation with tinnitus distress
Meyer et al. 2016^131^	MRI (SBM)	negative with gray matter volume bilateral supratemporal and cortical surface area of L periauditory cortex and anterior insula	–	correlation with tinnitus distress
Gunbey et al. 2017^132^	MRI (DTI)		fractional anisotropy between amygdala and hippocampus	negative correlation with tinnitus distress
Chen et al. 2017^133^	rs fMRI	–	L amygdala with L superior temporal gyrus; R amygdala with RL superior temporal gyrus	correlation with tinnitus distress
Mohan et al. 2018^134^	EEG	reduced temporal variability in parahippocampus	–	correlation with tinnitus distress
Besteher et al. 2019^135^	MRI (VBM)	no significant differences	–	high-distress tinnitus patients compared to low-distress tinnitus patients
Paraskevopoulos et al. 2019^136^	MEG	–	decrease in efficiency and small worldness of the resting-state network	correlation with tinnitus distress
Mohan et al. 2020^137^	EEG	–	connectivity from L hippocampus to L sgACC, R inferior frontal gyrus to R temporal pole; L hippocampus to R sgACC	negative correlation with tinnitus distress
Shahsavarani et al. 2021^138^	rs fMRI	–	higher connectivity in a network comprising cerebellum, precuneus, superior/middle frontal gyrus, primary visual cortex, dorsal attention network, salience network, and amygdala	high-distress tinnitus patients compared to low-distress tinnitus patients
Yoo et al. 2021^139^	rs fMRI	–	negative with path length; positive with clustering coefficient, small-worldness, and efficiency of information transfer	correlation with tinnitus distress
Ahmed et al. 2021^140^	MRI (DTI)	–	positive with L arcuate fasciculus density and radial diffusivity; negative with L arcuate fasciculus fractional anisotopy	correlation with tinnitus distress
Rosemann et al. 2023^141^	rs fMRI	–	connectivity between the precuneus and the lateral occipital complex	correlation with tinnitus distress
Elmer et al. 2023^142^	MRI (SBM)	cortical surface area of right dorsal prefrontal cortex and right posterior superior temporal sulcus	–	correlation with tinnitus distress
Vanneste et al. 2024^102^	EEG	increased alpha activity in dACC	–	correlation with tinnitus distress
Qin et al. 2025^143^	MRI (source-based morphometry)	negative with medial prefrontal cortex, precuneus, and auditory cortex	–	correlation with tinnitus distress

EEG, electroencephalography; fMRI, functional magnetic resonance imaging; rs fMRI, resting-state fMRI; MEG, magnetoencephalography; QEEG, quantitative EEG; VBM, voxel based morphometry; FDG PET, fluorodeoxyglucose PET; postACC, posterior ACC; SBM, surface based morphometry; DTI, diffusion tensor imaging.

The results of these studies show considerable variability, which likely reflects several methodological challenges. These include heterogeneity in imaging modalities and protocols (e.g., structural MRI, resting-state fMRI, and PET), differences in preprocessing and analytic pipelines, and variation in the clinical characterization of tinnitus and tinnitus distress. In addition, many studies are based on relatively small sample sizes, which may limit statistical power and reproducibility, and the use of multiple-comparisons correction is not uniform across the literature. Finally, most available studies are cross-sectional rather than longitudinal, which limits causal inference and makes it difficult to determine whether observed brain alterations represent predisposing factors, consequences, or correlates of tinnitus distress. Despite these limitations, the literature overall suggests that tinnitus distress is associated with greater involvement of non-auditory structures such as anterior cingulate, anterior insula, amygdala, (para)hippocampus, frontal cortex, precuneus and parietal cortex, as well as altered connectivity between these areas and auditory/temporal brain regions.

Altogether neuroimaging studies provide clear evidence that the brain structure, brain activation patterns, and brain connectivity differ between people who just perceive a phantom sound (“tinnitus”) and those who are suffering from their phantom sound (“tinnitus disorder”) and that the differences are consistent with additional involvement of a medial pathway linked to tinnitus-related distress in tinnitus disorder. Yet, it has to be acknowledged that these differences are based on tinnitus questionnaires that differentiate between high and low distress, which are assumed to correlate with tinnitus versus tinnitus disorder.

## Clinical, demographic, etiologic, and comorbid aspects of tinnitus and tinnitus disorder

### Cluster analyses identify tinnitus distress as a decisive factor for subtyping

Various approaches have been made to address tinnitus heterogeneity by identifying tinnitus subtypes by cluster analyses based on demographic and clinical information.^144^^,^^145^^,^^146^ In all these studies, tinnitus severity has been a distinctive factor between clusters. Tyler et al. differentiated patients into 4 clusters, with one cluster representing highly distressed patients and three further clusters of less distressed patients, which differed mainly in the influence of external factors on tinnitus (e.g., sound and somatic factors). Niemann et al. (2020) investigated 1,228 patients who underwent a 7-day treatment program. One cluster with over a half of the patients (*n* = 697) was characterized by low tinnitus distress, one cluster by high distress (*n* = 171), whereas the other two clusters (*n* = 163; *n* = 187) were in-between. Interestingly this pattern was found for all investigated health burden dimensions (tinnitus distress, general stress, physical quality of life, mental quality of life, internal resources, affective symptoms, somatic symptoms, and pain) but not for perceptual characteristics of tinnitus (tinnitus frequency, tinnitus localization/laterality, and type of tinnitus sound), where the clusters did not differ from each other. Reduction of the original 64-dimensional space into a two-dimensional projection of tinnitus patients confirmed the difference in health burden between the low-distress cluster and the other three clusters (Niemann et al. 2024). Thus, available data-driven cluster analyses of tinnitus patients confirm that among all clinical and demographic characteristics, tinnitus-related distress is the distinctive criterion for subtyping tinnitus patients.

### Stress and tinnitus

Physiological stress can be defined as an unpleasant sensory, emotional, and subjective experience that is associated with potential damage to body tissue and bodily or mental threat,^147^ especially when the environmental demand exceeds the natural regulatory capacity of an organism.^148^ Stress induces an immediate, adaptive, short-lived autonomic response and slower protracted activation of the hypothalamic-pituitary adrenal endocrine axis.^149^

In tinnitus, stress mediates the relationship between tinnitus loudness perception and tinnitus distress, i.e., loud tinnitus is predominantly distressing under stress.^122^ Mechanistically, stress results in locus coeruleus activation,^150^ releasing noradrenaline, which may modulate alpha-2 adrenergic receptors in the auditory cortex^150^^,^^151^ and the cochlear nucleus,^151^altering the sensory gain^152^ and consequently causing louder tinnitus perception under stress. Consistent with this, tinnitus patients tend to report louder and more bothersome tinnitus when they are stressed, although stress is not universally linked to tinnitus onset or severity.^153^ Neuroticism further amplifies the effects of stress, as neuroticism is associated with higher stress sensitivity and has consistently been associated with tinnitus severity and distress.^47^^,^^55^ Tinnitus catastrophizing, characterized by threat magnification, helplessness, and rumination,^154^^,^^155^ may represent a mechanism through which neuroticism exerts its effect on tinnitus-related suffering.

While acute sympathetic activation improves auditory function,^156^ chronic stress may worsen hearing.^157^ The sympathetic system regulates cochlear blood flow and modulates cochlear efferent fibers to alter the sensory gain. In the central auditory pathways, norepinephrine is essential for plasticity in the auditory cortex and affects the auditory cortex activity. Furthermore, long-term cortisol exposure becomes maladaptive, inducing comorbidities such as depression,^158^^,^^159^ anxiety,^159^ and insomnia,^160^ characteristics of tinnitus disorder.

### Psychoacoustic measures versus subjective measures

A consensus has emerged that neither loudness matching nor other psychoacoustic measures (pitch matching, maskability, and residual inhibition) of tinnitus bear a consistent relation with the severity or perceived loudness of tinnitus.^161^^,^^162^^,^^163^^,^^164^ Furthermore, no brain signatures can be identified that correlate with tinnitus matching or tinnitus pitch,^165^ whereas subjective NRS or VAS scores do correlate with activity in brain areas such as the auditory cortex, parahippocampoal area, insula, and ACC.^165^^,^^166^^,^^167^ This suggests that so-called more objective psychoacoustic measures cannot be used as outcome measures in tinnitus management or clinical trials.^164^

### Risk factors for tinnitus and tinnitus disorder

In a recent systematic review and meta-analysis of risk factors for tinnitus, hearing loss was confirmed as the most consistent major risk factor for tinnitus.^168^ There was only low-level evidence for further risk factors.^168^ In contrast, among patients with bothersome tinnitus, various non-auditory risk factors for tinnitus severity could be identified, including neuroticism, mood disorders, pain syndromes, and sleep problems.^47^^,^^48^^,^^54^^,^^57^^,^^169^^,^^170^^,^^171^^,^^172^^,^^173^^,^^174^^,^^175^

An explanation for this discrepancy is provided by a recent study in which data from the UK biobank were used to identify risk factors for “tinnitus” (tinnitus presence) and “tinnitus disorder” (tinnitus severity) separately.^55^ Moreover, it was analyzed that to what extent these risk factors predict the evolution of tinnitus to tinnitus disorder over time. The results revealed a dissociation between features predicting tinnitus presence and tinnitus severity. While hearing health emerged as a common key predictor of presence and severity, other factors such as mood, neuroticism, and sleep only predicted its severity. Interestingly, while the “presence” model did not predict the evolution of tinnitus over time, the “severity” model provided an estimation of its progression over nine years, with a large effect size for individuals likely to develop severe tinnitus.

The identification of specific risk factors for tinnitus suffering and its progression over time nuances the notion that tinnitus-related suffering is a “normal” reaction to a disturbing signal and, therefore, should neither be considered as a pathology nor be specifically diagnosed.^34^ While transient suffering may be normal, persistent suffering may be pathological, analogous to persistent suffering after a loss, which is then called persistent grief.^176^ Consequently, transient mourning for silence,^177^ which is lost in tinnitus, is normal, but persistent grief may be pathological and result in a tinnitus disorder. However, if the suffering persists, this is more indicative of a different trajectory rather than a personal or pathological failure and requires appropriate clinical support as well as, consequently, a corresponding definition in the ICD and DSM codes. There may be people who suffer from the onset of tinnitus and do not show improvement, whereas others may improve over time.^177^^,^^178^^,^^179^^,^^180^ The first group may have tinnitus disorder from the beginning, whereas the second group may be in keeping with “normal” suffering^34^ that improves over time.^178^^,^^179^^,^^180^ This would also suggest that tinnitus disorder must be defined only in chronic tinnitus and not in acute tinnitus. Most people may never show tinnitus-related suffering, and rarely do people not suffer initially but start suffering at a later stage. These 4 trajectories may be similar to the trajectories described in grief.^176^^,^^177^^,^^181^ A small pilot study revealed that 20% of patients who are initially bothered by their tinnitus are less so after some months, seeing a reduction of 20 points on the Tinnitus Handicap Inventory (THI) scale, and almost 50% see a reduction of 7 points,^182^ which is the minimal clinically important difference (MCID) for the THI.^183^ This is in keeping with what has been theorized based on data in bereavement.^177^

In summary, hearing function is a risk factor for tinnitus presence, whereas tinnitus severity and the progression of severity over time are additionally determined by personality (neuroticism), mood, sleep, and fatigue,^55^ whereas no associations have been found with psychoacoustic measures of tinnitus.

### Therapeutic interventions can reduce tinnitus suffering without changing the tinnitus percept

Except for cochlear implants^184^^,^^185^^,^^186^^,^^187^^,^^188^ and auditory cortex implants,^189^ no treatments can systematically reduce tinnitus loudness. In other words, no approved treatments exist for tinnitus.

On the other hand, various treatments can reduce the severity of tinnitus disorder by reducing tinnitus suffering without altering the tinnitus loudness, demonstrating that these pathways are separable. This was already recognized during frontal lobotomies performed as the treatment of chronic tinnitus.^190^^,^^191^ In 17 patients who underwent a prefrontal leucotomy for tinnitus, “With regard to the quality of the improvement: no patient became free from tinnitus, many still maintained that the noises were as bad as ever, yet in some way every patient was able to adjust better to his noises. This appeared mainly as an improvement in mood and as a reduction in the amount of attention paid to the tinnitus which had become, as it were, shifted toward the periphery of consciousness”.^191^ Thus, the suffering component of tinnitus can be improved without improvement in loudness, which is in keeping with the meta-analysis results from various tinnitus treatments including cognitive behavioral therapy (CBT),^192^ neuromodulation via repetitive transcranial magnetic stimulation (rTMS),^193^^,^^194^^,^^195^ transcranial direct current stimulation (tDCS),^196^^,^^197^ as well as acupuncture^198^ for tinnitus. They all revealed an improvement in suffering, as measured by tinnitus questionnaires, without improvement in tinnitus loudness. Cochlear implants, the only treatment that can reliably reduce tinnitus loudness, has also beneficial effects on tinnitus handicap,^187^ indicating that silencing tinnitus also reduces tinnitus suffering.^184^^,^^185^^,^^186^^,^^187^^,^^188^

### Future of tinnitus and tinnitus disorder

From a patient perspective, a dimensional, individualized approach may be superior to a categorical health care provider approach. The dimensional model may outperform a categorical approach if patient management is shifted from a clinical to a neurobiological approach, but this requires developing a taxonomy that links each dimension to a specific brain correlate that can be addressed selectively. Thus, for example, if tinnitus loudness can be related to a specific network, anxiety to another network, depression to still another, cognitive deficits to yet another network and that each of these networks can be identified in an individual patient, then a dimensional approach is ideal. As long as these objective signatures cannot be identified, a categorical approach may be more pragmatic to implement. Yet, a hybrid system can be proposed that creates subcategories, e.g., grading of tinnitus disorder into mild, moderate, and severe grades. This requires the thresholds or cut-offs to be determined for tinnitus versus tinnitus disorder and the cut-offs to be developed for the different tinnitus disorder grades. For tinnitus disorder grading, generally accepted tinnitus questionnaires can be used that already propose grading into mild, moderate, and severe. Further confirmation can be found in correlations with tinnitus-unrelated questionnaires that evaluate the severity of suffering in similar grades (mild, moderate, and severe) for anxiety (e.g., Hospital Anxiety and Depression Scale [HADS]) or depression (e.g., HADS and Beck's Depression Inventory [BDI]). Consequently, for only tinnitus to be present and not tinnitus disorder, tinnitus questionnaires like Tinnitus Questionnaire (TQ), THI, and Tinnitus Functional Index (TFI) would indicate values consistent with a compensated state, while scores on the HADS, BDI, or other suffering-related questionnaires would fall within normal population range. Currently no consensus exists on the categorical separations, but based on the above reasoning, a table can be developed as a proposal for defining categorical distinctions between tinnitus and tinnitus disorder and between tinnitus disorder severity grades (Table 2).

**Table 2:** Distinguishing between tinnitus and tinnitus disorder based on questionnaire ratings

			Tinnitus	Tinnitus disorder		
Scale	Assessed concept	Scale range	Normal	Mild	Moderate	Severe
HADSa	anxiety	0–21	<8	8–10	11–14	15–21
HADSd	depression	0–21	<8	8–10	11–14	15–21
BDI	depression	0–63	<14	14–19	20–28	29–63
TQ	tinnitus distress	0–84	<31	31–46	47–59	60–84
THI	functional impact of tinnitus	0–100	<18	18–36	38–56	58–100
TFI	functional impact of tinnitus	0–100	<25	25–50	50–75	75–100

One could go a step further and simplify or compress clinical management even more by looking for numeric rating scale (NRS) or visual analogue scale (VAS) scores that correlate with mild, moderate, and severe suffering. In pain research, it has been demonstrated that there may exist 2 thresholds, based on a cluster analysis of a relatively large group (*n* = 163) of patients presenting at a neurosurgical clinic for pain treatment.^199^ Three statistically different groups were identified. The first group had a VAS score of ≤5, with both normal suffering and disability questionnaires. These patients only had painfulness and neither suffering nor disability. The second group had VAS scores of ≥6, with abnormal suffering questionnaires but no disability, i.e., they had painfulness and suffered but were still functioning normally. The third group had VAS scores of ≥7, with abnormal suffering and abnormal disability questionnaires.^199^ A similar study in tinnitus may benefit the distinction between tinnitus and tinnitus disorder and may further suggest a grading in tinnitus disorder, analogous to what is done in, for example, substance use disorder, in DSM-V in which 3 grades exist: mild, moderate, and severe. This grading is determined by how many of 11 questions are answered in a confirmatory way: ≤3, mild substance use disorder; ≤5, moderate substance use disorder; and ≥7, severe substance use disorder.

In summary, the cut-offs will be important to correctly distinguish between tinnitus and tinnitus disorder, as well as to grade tinnitus disorder. This will be instrumental for a better understanding of the pathophysiology of tinnitus, for example, to distinguish the tinnitus sound network from the suffering and the disability network. However, this is also important for epidemiological studies, genetic profiling, and develoment of therapies that target each of these components (sound, suffering, and disability), thereby enabliing personalized treatments.

Considering that suffering in acute tinnitus may be normal and that suffering reduces in time, it may be important to define tinnitus disorder only after a minimum duration. Based on an analogy with chronic pain, an arbitrary duration of 3 months has been proposed to define tinnitus as chronic.^1^ Whether 3 months would qualify as a minimum duration to define tinnitus disorder needs to be seen. The minimum duration could be determined based on data rather that consensus by evaluating how long is the average time for tinnitus recovery, as described by the four trajectory models.^177^

## Conclusion

This perspective argues for a clear categorical distinction between tinnitus and tinnitus disorder for pragmatic reasons, supported by genetic, anatomical, pathophysiological, neuro-imaging, and clinical evidence. While tinnitus is the perception of a sound without any external source, tinnitus disorder involves the perception of a phantom sound coupled with suffering and disability. This distinction is facilitating scientific research as the two entities differ in their genetic underpinnings, the involved brain networks, and their clinical risk profile. Distinguishing between tinnitus and tinnitus disorder better accounts for the clinical heterogeneity of the affected patients and is crucial for developing targeted and effective therapies for each condition. Further development of strict diagnostic criteria that differentiate tinnitus from tinnitus disorder as well as a grading system that details the severity of tinnitus disorder is mandated.

## Declaration of interests

The authors declare no competing interests.

## Declaration of generative AI and AI-assisted technologies in the writing process

No AI tool was used to generate any part of the paper.
